# A Case of Erysipelas

**Published:** 1909-07

**Authors:** G. B. Stocker

**Affiliations:** Buffalo, N. Y.


### A Case of Erysipelas

BY G. B. STOCKER, M. D., Buffalo, N. Y.

AMONG the three hundred or more mal-conditions which the practitioner is called upon to treat, there are few which tax his skill more severely than erysipelas. Because of the rapidly progressive and overpowering nature of the infection—its tendency to spread over a larger and larger area of skin surface and to surcharge the system with its toxins—treatment must be administered with dispatch. In a large proportion of cases erysipelas develops because the vital resistance of the patient has been lowered by disease or operation; for this reason the situation is particularly serious—this lowered resistance being a handicap to the patient which may easily prove disastrous.

Therefore, the value of supportive measures becomes apparent. Absolute rest in bed, a nourishing diet, and stimulants of one kind or another are indicated. And later, during convalescence, bitter tonics do good service when recovery of strength is slow. Of the numerous special remedies recommended for erysipelas the one that has enjoyed the most favor is the tincture of ferric chloride. One writer speaks of it as follows: "The internal treatment of this disease *par excellence* is the plentiful use of the tincture of the chloride of iron—twenty to thirty minims—well diluted, four times a day." While apparently of some service, this drug is certainly not a specific for the disease and should be withdrawn if it disturbs digestion. The therapeutic status of antistreptococcus serum has not yet been definitely determined, but it has frequently been administered in severe cases with good results.

The application of ichthyol is considered by many practitioners the best local treatment of this disease. It is usually employed in the form of an ointment—20 to 30 per cent.—in lanolin. I have used ichthyol in past years but more recently have taken up the organic peroxide. In the case under consideration this agent proved successful after ichthyol had failed to accomplish good results.

An undertaker who had recently buried a man who died of erysipelas developed, two days later, a bad case of the disease. I was called in and treated the condition locally with ichthyol. A few days after my first visit I was hurriedly called and found the patient with a temperature of 106° and a fresh infection, beginning at the collar line and extending over the entire back, and, also, in streaks, from the shoulder down the chest. The patient could no longer tolerate the odor of ichthyol, he informed me, so, after consultation with Dr. Frank McGuire, who was called in, we decided to try acetozone. We made a solution of the drug—10 grains to one quart of hot water—and applied compresses dipped in this solution as hot as could be borne. These were changed every twenty or thirty minutes and continued all night; in the morning the temperature had fallen four degrees and the sick man was much improved. This treatment was kept up for thirty-six hours. In addition, a mixture of acetozone and collodion was painted on the streaks and along the borders, which procedure seemed to check the infection and to prevent its spread. The patient made an uneventful recovery.

Others have reported equally good results from acetozone after applications of mercury bichloride, ichthyol and other remedies had failed to check the progress of the disease. I believe this drug deserves to be more frequently employed in erysipelas.

901 Genesee Street.

